# Streptococcus Mutans: A Potential Risk Factor in Recurrent Hemorrhagic Stroke

**DOI:** 10.7759/cureus.1264

**Published:** 2017-05-19

**Authors:** Tarek R Mansour, Yasaman Alam, Layth Dahbour, Ahmed Alnemari, Mouhammad Jumaa, Jason L Schroeder

**Affiliations:** 1 Department of Surgery, Division of Neurosurgery, The University of Toledo Medical Center; 2 Surgery / Division of Neurological Surgery, The University of Toledo Medical Center; 3 Department of Neurology, The University of Toledo Medical Center; 4 Department of Surgery, The University of Toledo Medical Center

**Keywords:** collagen binding protein, chronic hypertension, hemorrhagic stroke, intracerebral hemorrhage, suboccipital craniotomy, streptococcus mutans, cbp, s. mutans

## Abstract

Stroke is the fifth leading cause of death and is responsible for approximately nine percent of all deaths worldwide. Cases of Streptococcus mutans (S. mutans*)*-induced intracerebral hemorrhage as a result of bloodstream infections have seldom been reported. New reports show that bacteria with specific collagen binding proteins (CBPs), such as the Cnm type produced by S. mutans, may inhibit platelet aggregation and cause bleeding.

In this article, we report on a 62-year-old man with a recent history of left frontal intracerebral hemorrhage (ICH) who presented to the emergency department after a fall due to suspected seizure while in rehabilitation. Computed tomography (CT) scan of the brain showed a right cerebellar hemorrhage with surrounding edema and mass effect on the fourth ventricle. A suboccipital craniotomy to evacuate the cerebellar ICH was completed without complication. Radiologic and angiographic assessments regarding the etiology of this patient’s stroke did not reveal any evidence of vascular pathology or mycotic aneurysms to explain his recurrent intracranial hemorrhages. Through persistent patient and family interviews, it came to light that a few weeks prior to the patient’s first ICH, he was diagnosed with a bloodstream infection by S. mutans*.*

Bacteremia is known to be associated with embolic stroke, but only recently has it been shown that bacteremia can also be implicated in hemorrhagic stroke. S. mutans of the k serotype have specific CBPs that are attracted to exposed collagen in previously damaged small vessel walls. These bacterial proteins can interrupt the blood clotting cascade through the prevention of platelet aggregation, increasing the risk of intracerebral hemorrhage.

## Introduction

Stroke is the fifth leading cause of death and is responsible for approximately 9% of all deaths worldwide [1]. Stroke can be classified as ischemic, which accounts for 85% of stroke cases, or hemorrhagic, accounting for the remaining 15% of cases [2]. Several risk factors, such as hypertension, history of ischemic stroke, brain malignancy, diabetes mellitus, and hyperlipidemia, have been identified for stroke, but studies have shown that a large number have no clear cause [3]. Given the sudden onset of intracerebral hemorrhage (ICH), its mortality risk, and the potential for debilitating neurological complications, understanding potential uncommon etiologies is essential to improving the care, management, and prevention of these stroke types. Identifying uncommon stroke etiologies requires a comprehensive interview with the patient/family and documentation of the patient’s past medical history. Further, documenting the circumstances surrounding the onset of the stroke, its chronology, and evolution help to ultimately understand if there is a potential unusual risk factor associated.

In the past, Streptococcus mutans (S. mutans), a group of viridans streptococci commonly found among the oral flora, has been implicated in several cases of infective endocarditis following dental procedures; however, cases of S. mutans-induced intracerebral hemorrhage as a result of bloodstream infections have seldom been reported. Reports related to infective endocarditis have described the potentially implicated mechanism as being the induction of platelet activation and aggregation by various bacteria; however, new reports show that bacteria with specific collagen binding proteins (CBPs), such as the Cnm type produced by S. mutans, may inhibit platelet aggregation and cause bleeding [4-6].

It has been proposed that S. mutans with CBPs, namely, of the Cnm type, can interrupt the interaction between exposed collagen fibers on damaged endothelium and platelet surfaces, effectively disrupting the initiation of platelet aggregation and inhibiting hemostasis. Furthermore, Cnm-positive S. mutans has been shown to potentially activate matrix metalloproteinases that lead to further disruption of blood vessel barriers and bleeding. The current literature reports on S. mutans-induced intracerebral microbleeds but does not provide understanding regarding its implications in delayed ICH and recurrent hemorrhage [5-7]. This case report provides the history of a patient with delayed, recurrent, and distantly located intracranial hemorrhagic episodes after documentation of prior ­S. mutans septicemia. Understanding the potential relationship between S. mutans blood stream infection and intracranial hemorrhage could alter patient education and management during such infections in an effort to prevent delay in diagnosis for patients exhibiting stroke symptoms. Early diagnosis and treatment of infection with S. mutans can potentially reduce the risk of morbidity and mortality related to post-septicemia ICH. 

## Case presentation

**Figure 1:**
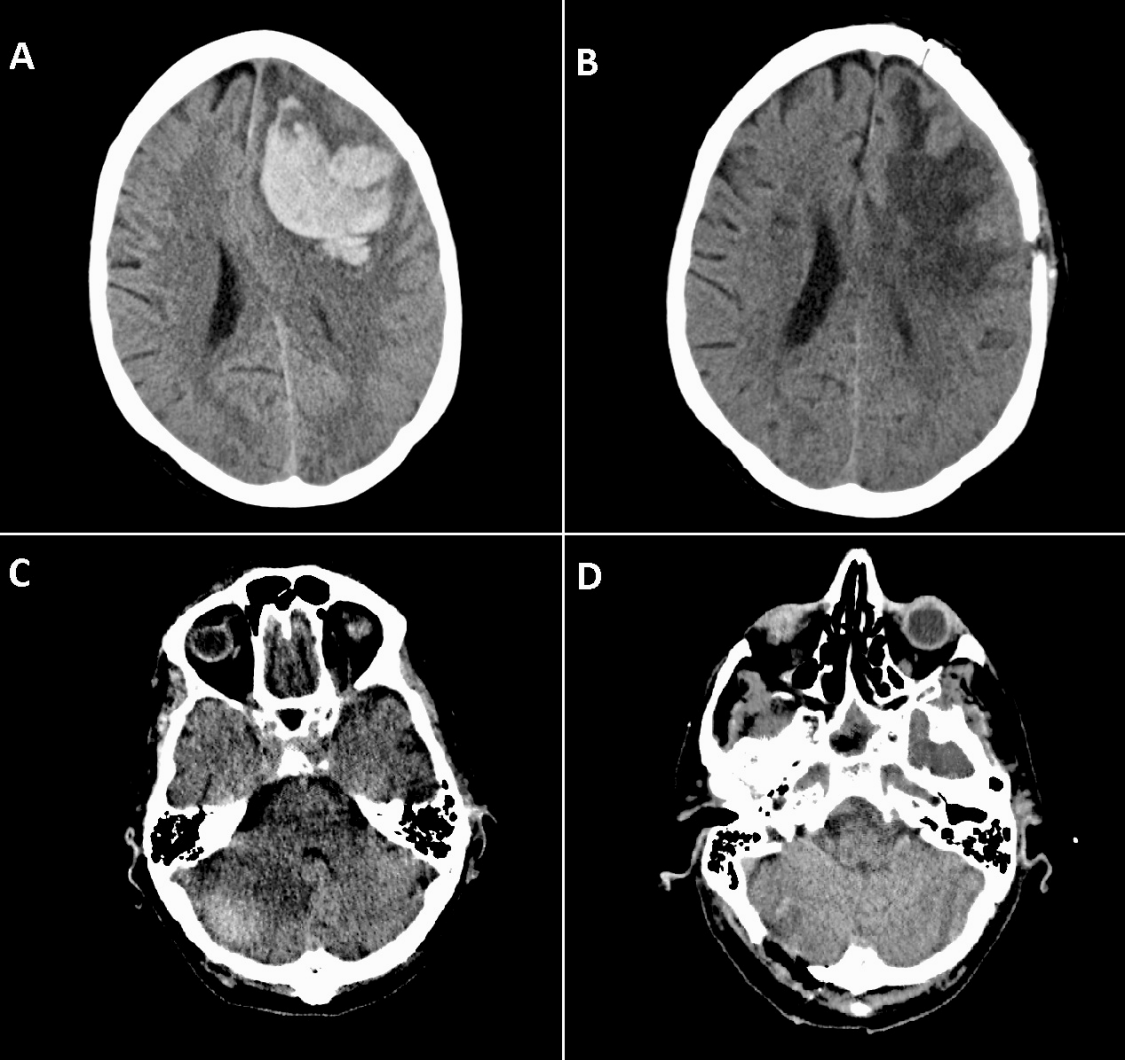
Computed tomography (CT) scans of the patient’s brain (A) Left frontal intracerebral hemorrhage (ICH) preoperatively; (B) Left frontal ICH postoperatively; (C) Right cerebellar ICH preoperatively; (D) Right cerebellar ICH postoperatively.

The patient is a 62-year-old male with a recent history of left frontal ICH (Fig. 1A, 1B), hypertension, and atrial fibrillation (not anticoagulated) who presented to the emergency department after a fall due to suspected seizure while in rehabilitation. There was no other history of risk factors related to ischemic or hemorrhagic stroke. At presentation, the patient was unconscious and intubated with pupils that remained reactive to light. Computed tomography (CT) scan of the brain showed a right cerebellar hemorrhage with surrounding edema and mass effect on the fourth ventricle (Figure 1C, 1D). The patient’s family was counseled and, after review of the potential risks and benefits, consented to the surgical evacuation of the cerebellar ICH.

A suboccipital craniotomy was completed without complication. His hospital course was complicated by Klebsiella pneumoniae pneumonia, and despite treatment for this, he required re-intubation and subsequent tracheostomy and gastrostomy placement due to failure to progress. He was later discharged back to rehabilitation in improved condition.

**Figure 2:**
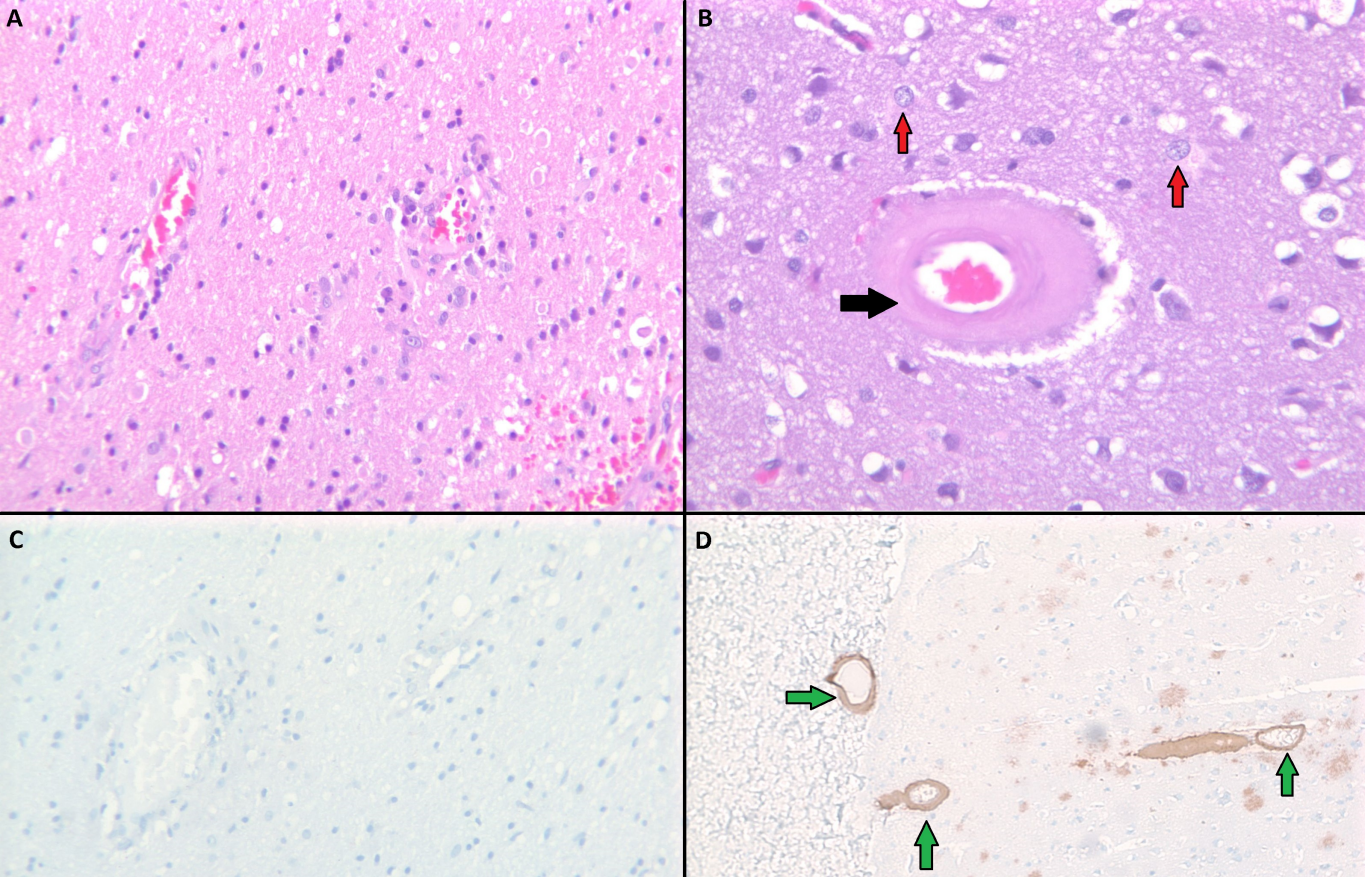
Pathology images of negative brain biopsy results from the patient’s brain (A and C) and positive control (B and D). (A) Hematoxylin and eosin (H&E) negative for vasculitis; (B) H&E positive control showing thickening of the vessel wall (black arrow) with surrounding inflammatory infiltrates (red arrows); (C) Negative immunohistochemical staining against amyloid-β; (D) Positive control showing deposition of amyloid-β in vessel walls (green arrows).

Radiological assessment regarding the etiology of this patient’s stroke included multiple CT scans, magnetic resonance imaging (MRI) of the brain, including MR angiography of the head and neck, and cerebral angiography. The CT scans demonstrated his prior left frontal ICH and his new right cerebellar ICH at this presentation. His MRI scan did not show any acute ischemic stroke in the cerebellum associated with the new ICH. His cerebral angiography was unremarkable and did not reveal any evidence of vascular pathology or mycotic aneurysms to explain his recurrent intracranial hemorrhages. His blood workup at presentation showed normal coagulation studies and his platelet count was low normal at 119 x 10^9^/L. Post-resection pathology report showed acute intracranial hemorrhage but special stains did not reveal any evidence of amyloid angiopathy or vasculitis (Figure 2). Through persistent patient and family interviews, it came to light that a few weeks prior to the patient’s first ICH, he was diagnosed with a bloodstream infection by S. mutans.

## Discussion

Stroke is defined by the World Health Organization as the sudden and rapid development of neurological signs indicating “focal or global disturbance of cerebral activity, lasting for more than 24 hours or leading to death, with no apparent cause other than that of a vascular origin” [2]. Given that stroke is a leading cause of death, interest in and the scope of research related to stroke has grown significantly over the past few decades. Even though intracerebral hemorrhages account for only 15% of all strokes, they are responsible for 40% of stroke-related deaths [1, 3].

Hemorrhagic stroke can lead to disability or death, and due to this potential for poor outcomes, it is important to recognize and treat any identifiable underlying risk factors early in an effort to reduce the risk of sustaining ICH. The literature suggests that risk factors for hemorrhagic stroke include chronic hypertension, diabetes, cerebral aneurysm, coagulopathy, arteriovenous malformation (AVM), anticoagulant usage, traumatic brain injury, obesity, smoking, excessive alcohol intake, and unhealthy diet [3]. This patient had only hypertension, which could prospectively be considered a clear, identifiable risk factor for ICH. However, the subsequent discovery of prior S. mutans septicemia raises the possibility that this blood stream infection may also potentially be implicated as a risk factor for the development of recurrent hemorrhages in this patient.

S. mutans, a gram-positive viridans streptococci, has four serotypes, c, e, f, and k, and is frequently associated with the development of dental caries. In this setting, 80% of the isolates are of the c serotype and only 5% of the isolates are of the k serotype [6-8]. In contrast, S. mutans of the k serotype is the only isolate that has been associated with hemorrhagic stroke. In a study carried out by Nakano, et al., it has been shown that k serotype S. mutans can produce a collagen-binding protein that interferes with platelet aggregation and inhibits the formation of platelet plugs, leading to hemorrhage [6]. Furthermore, a study by Miyatani, et al.* *was conducted in which 51 subjects infected with S. mutans capable of producing CBP of the Cnm type were observed and tested for cerebral microbleeds. Out of the 51 subjects who had a bloodstream infection with Cnm-positive S. mutans, 43 had evidence of cerebral microbleeds on MRI [5]. These strains of S. mutans are attracted to collagen and, therefore, are most likely to be found in previously damaged vasculature where collagen is exposed [6].

Chronic hypertension was also a risk factor for ICH in this patient. After his first ICH, the patient was discharged with a systolic blood pressure goal of less than 140 mmHg using an oral calcium channel blocker. Although the pathogenesis of endothelial dysfunction and hypertension has been widely studied, the directionality of the relationship between them has been controversial. Hypertension has been linked to endothelial dysfunction through the generation of reactive oxygen species and inflammation. Chronic hypertension causes degeneration of small arteries, leading to microaneurysms which can potentially rupture. Additionally, the permeability of the blood-brain barrier (BBB) decreases with age and is at least partially due to the loss of endothelial integrity [9]. Longstanding hypertension further affects the structure of small vessels, leading not only to BBB disruption but also to the accumulation of Type I collagen in the walls of small vessels [10]. This increase in collagen within the damaged vessel walls may again serve as a substrate for the deleterious effects of S. mutans CBPs [6, 9].

In the case presented, it is plausible that the patient had a variety of interplaying risk factors, which ultimately led to the development of intracerebral hemorrhage. However, the development of two distantly located hemorrhagic strokes within a two-month time period should prompt investigation for additional and unusual ICH risk factors. 

## Conclusions

S. mutans of the k serotype have specific CBPs that are attracted to exposed collagen in previously damaged small vessel walls. These bacterial proteins can interrupt the blood clotting cascade through the prevention of platelet aggregation, increasing the risk of intracerebral hemorrhage. In addition to CBPs, S. mutans can produce metalloproteinases that are capable of digesting elastin in arterial walls, which may also contribute to the disruption of blood vessels and result in bleeding.

Infective endocarditis and bacteremia are known to be associated with embolic stroke, but only recently has it been shown that bacteremia can also be implicated in hemorrhagic stroke. Therefore, clarifying the level of risk involved with S. mutans septicemia and its potential association with intracerebral hemorrhage in intermediate and high-risk patients can lead to better strategies for counseling and monitoring patients post-infection. Pursuing such measures can potentially reduce the morbidity and mortality associated with primary or recurrent intracranial hemorrhages. 
